# Paratuberculosis in a domestic dog in South Africa

**DOI:** 10.4102/jsava.v88i0.1441

**Published:** 2017-03-30

**Authors:** Michele A. Miller, Sewellyn C. Davey, Lesley S. van Helden, Frank Kettner, Sandy M. Weltan, Rick Last, John D. Grewar, Louise Botha, Paul D. van Helden

**Affiliations:** 1 DST NRF Centre of Excellence for Biomedical Tuberculosis Research, South African Medical Research Council Centre for Tuberculosis Research, Division of Molecular Biology and Human Genetics, Stellenbosch University, South Africa; 2 Department of Agriculture, Western Cape Veterinary Services, South Africa; 3 Tygerberg Animal Hospital, Bellville, South Africa; 4 Vet Diagnostix Cape Town, Bellville, South Africa

## Abstract

This case report shows that *Mycobacterium avium* subsp. *paratuberculosis* (MAP) infection can cause clinical disease in domestic dogs, and should be considered as a differential diagnosis for gastrointestinal inflammatory conditions. A male dachshund presented with lethargy and pain. Enlarged mesenteric lymph nodes were found on abdominal ultrasound examination. Cytological examination of lymph node aspirates was consistent with granulomatous inflammation, which was culture-confirmed as MAP. Although we were unable to confirm the source of infection, the dog’s history included exposure to sheep in the Western Cape.

## Introduction

*Mycobacterium avium* subsp. *paratuberculosis* (MAP) infection is the cause of Johne’s disease (paratuberculosis) in domestic ruminants and wildlife worldwide (Manning 2001; Manning & Collins 2001; Stevenson et al. 2009). Johne’s disease, or paratuberculosis, is a chronic progressive intestinal disease resulting in thickening of the intestinal wall that impairs nutrient absorption and results in decreased productivity, diarrhoea and wasting. Once clinical signs of the disease develop, death is inevitable. Although most commonly it affects ruminants, other species including rabbits, feral cats and free-ranging carnivores have also been infected (Manning & Collins 2001; Matos et al. 2014; Palmer et al. 2005). Therefore, the host range of species infected is broader than ruminants. Gastrointestinal inflammation has many diverse causes in dogs and cats. In one study (Glanemann et al. 2008), 19% of dogs with chronic vomiting or diarrhoea were found positive for MAP-specific DNA in intestinal biopsies, suggesting that MAP may be an under-recognised cause of gastrointestinal disease in dogs. This case report describes the clinical signs, diagnosis, treatment and necropsy findings of a case of paratuberculosis in a domestic dog and emphasises the importance of including MAP infection in the differential diagnosis of splenomegaly, abdominal lymphadenopathy or granulomatous ileitis in companion animals.

## Ethical considerations

Informed consent was provided by the owner to the attending veterinarian for clinical procedures and sharing records.

## Diagnosis and management

A 2-year-old, 6.6 kg castrated male dachshund presented for routine vaccination, although the owner complained that the dog appeared to be listless. Within 24 hours, the dog became more lethargic with reduced appetite and appeared to be in pain when handled. Physical examination revealed pale mucous membranes and tachycardia with weak pulses. Although no pain was elicited on examination, the dog exhibited a stiff high-stepping gait in both hind limbs. Rectal temperature was 39.4 °C. The patient had normochromic, macrocytic, regenerative anaemia (haematocrit 25%, reticulocyte count 98 × 10^9^/L) and mild lymphocytosis (7.35 × 10^9^/L). No haemoparasites were found, and platelets appeared to be normal. Additional test results (saline agglutination, Coombs test and *Ehrlichia* Snap test) were negative. The patient was hospitalised, placed on intravenous fluids and administered 0.18 mg/kg dexamethasone (Dexafort, Intervet SA) subcutaneously and 20 mg/kg amoxicillin-clavulanic acid (Clavamox, Sandoz Co., Sandoz) intravenously and referred the next day.

**FIGURE 1:**
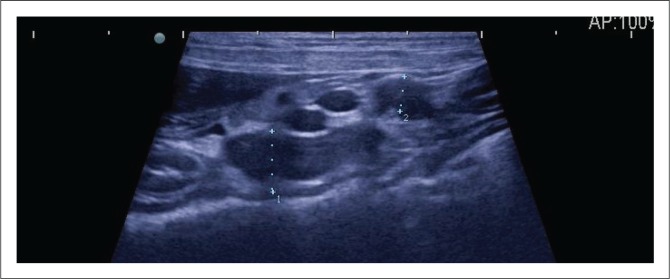
Abdominal ultrasound image of the enlarged mesenteric lymph nodes, indicated by callipers labelled 1 and 2 (measurements of 8 mm and 5 mm, respectively).

**FIGURE 2:**
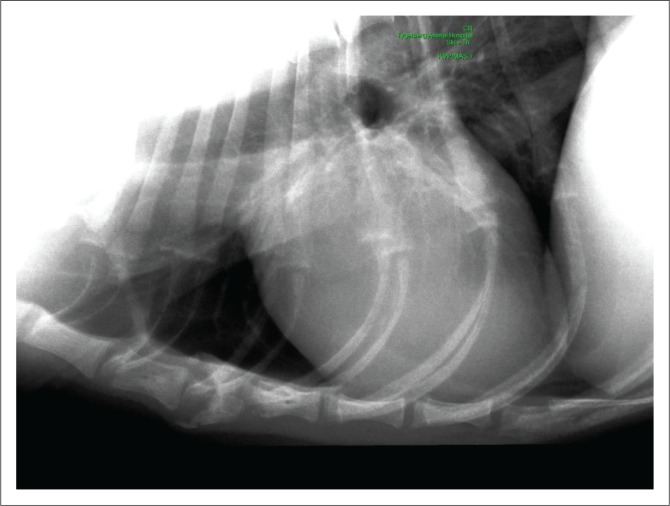
Radiographic lesion of the sternebrae (left lateral thoracic radiographs).

**FIGURE 3:**
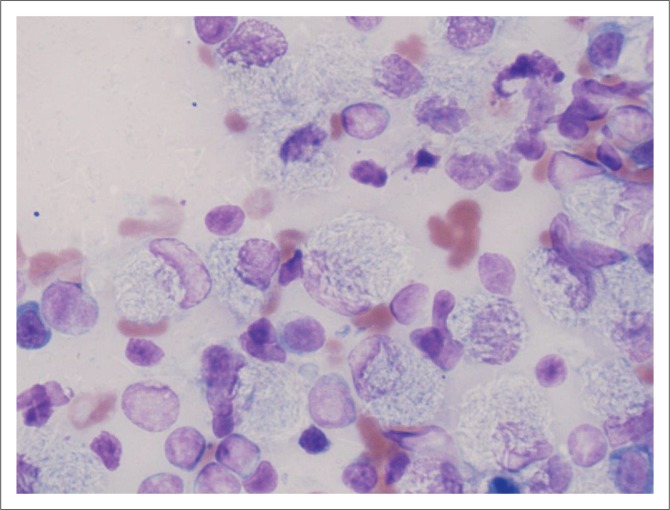
Wright-Giemsa stained mesenteric lymph node cytology (1000 x).

At referral, physical examination revealed no new findings, although the abnormal gait was resolved and heart rate and pulses normalised (heart rate 114 beats per minute). Rectal temperature was 39.4 °C. On occasions during the examination, the dog would exhibit pain that could not be localised (although this could have been a reaction to the recent vaccination). Clinicopathological investigations were repeated with, except for an increased serum C-reactive protein (46 mg/L [reference range less than 20 mg/L]), no significant changes. Abdominal ultrasound revealed enlarged mesenteric lymph nodes (Figure 1) and a subjectively enlarged spleen. Thoracic radiographs showed lytic lesions of the fifth and sixth sternebrae (Figure 2). Cytological analyses of fine needle aspirates of enlarged lymph nodes, spleen and sternebra demonstrated (Figure 3) a mixed population of inflammatory cells predominated by macrophages, with small and reactive lymphocytes, plasma cells and non-degenerate neutrophils in the spleen and lymph node aspirates. Most of the macrophages contained large numbers of negative-staining bacilli. The smears from the sternebra contained a similar but less severe infiltration of inflammatory cells but included negative-staining bacilli in macrophages. These findings were consistent with granulomatous inflammation associated with mycobacterial infection.

## Mycobacterial culture

Mesenteric lymph node and splenic tissues were cultured using mycobacterial growth indicator tubes (BD Biosciences, USA) together with the automated Bactec 960 TB system (BD Biosciences, USA) as previously described (Warren et al. 2006). To identify mycobacterial species, polymerase chain reaction (PCR) and sequencing of the 16S rRNA gene were done using 1.1 pmol/uL 16S rRNA forward primer (5’ – AGA GTT TGA TCC TGG CTC AG – 3’) at the Central Analytical Facility (CAF) of Stellenbosch University, South Africa (Katale et al. 2015). Accession numbers and obtained sequences were edited and analysed using the ribosomal differentiation of medical microorganisms (RIDOM) project (http://www.ridom-rdna.de) and the National Center for Biotechnology Information (NCBI) blast sequence alignment tool (http://blast.ncbi.nlm.nih.gov).

## Treatment and outcome

Treatment was initially started with doxycycline (75 mg twice daily) and enrofloxacin (50 mg orally once daily). This was changed approximately after 1 month when PCR results became available to oral rifampicin 75 mg once daily, oral clarithromycin 62.5 mg twice daily and oral doxycycline 75 mg twice daily. Supportive treatment with mirtazapine 3.75 mg once daily and ondansetron 4 mg twice daily was also prescribed. After 2 months, the patient had gained 1 kg, and there had been a noticeable improvement in its demeanour. Abdominal lymphadenopathy was unchanged on ultrasound examination. Results of repeated fine needle aspiration cytology of the mesenteric lymph nodes and spleen were similar to the samples collected at initial diagnosis. Negative-staining bacterial rods were again identified in both the spleen and abdominal lymph nodes. The sternebra was not resampled. The patient was discharged with an increased dosage of rifampicin (112.5 mg once daily) and clarithromycin (62.5 mg three times daily). Clofazimine (http://www.johnes.org/antimicro/index.html; Greene 2006), 50 mg daily, was added after approval was obtained from the Medicines Control Council.

Nine months after initial referral, the patient had lost 800 g body weight over the previous 5 months. Pyrexia was present, and mesenteric lymphadenopathy was still observed on abdominal ultrasound examination. Aspirate cytology was not repeated. Treatment was continued unchanged. Eleven months after initial referral, the patient was hospitalised for pyrexia, lethargy, general malaise, decreased appetite and progressive weight loss (body weight was now 6.1 kg). Lymphadenopathy and marked splenomegaly were noted on abdominal ultrasound. Cytology sampling was not repeated. The patient’s quality of life remained poor despite a brief attempt at palliative care, and euthanasia was advised and performed.

**FIGURE 4:**
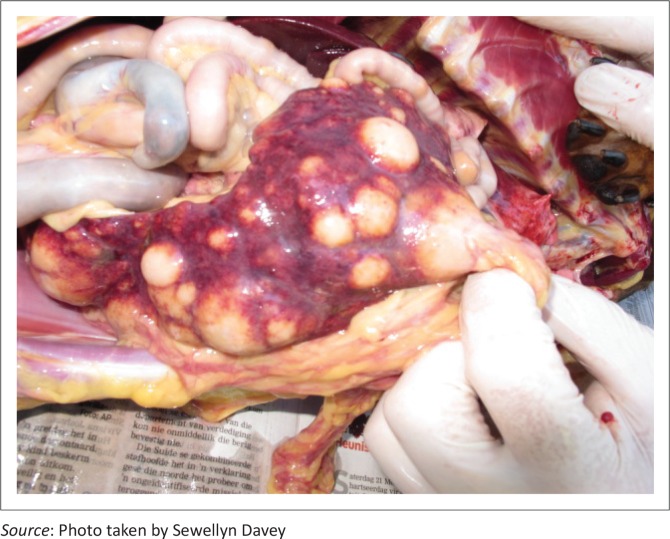
Multifocal lesions in spleen on post-mortem examination.

At post-mortem examination, the dog was thin and tissues appeared icteric, although the underlying cause was undetermined. The most notable changes were multifocal cream-coloured masses in the spleen, ranging in size from 1 mm to 50 mm in diameter (Figure 4). Histopathology revealed severe granulomatous enteritis with sheets of epithelioid macrophages and Langerhans giant cells, exclusively restricted to the submucosa of the ileum. Multifocal granulomatous lymphangitis was observed in the tissue adjacent to the inner layer of the tunica muscularis. There was multifocal nodular to coalescing granulomatous splenitis with effacement of normal splenic architecture by sheets of epithelioid macrophages. In addition, multifocal granulomatous interstitial nephritis was present. On Ziehl–Neelsen staining, epithelioid macrophages and Langerhans giant cells in the ileum, spleen and kidney were engorged with myriad acid-fast filamentous bacteria, consistent with infection by MAP.

## Discussion

MAP infection is a significant veterinary issue for cattle and sheep, and may be an under-recognised infectious disease of domestic and wild carnivores, especially in areas where Johne’s disease is present in livestock (Beard et al. 2001; Manning & Collins 2001; Stevenson et al. 2009). MAP was isolated from tissues of foxes (82%) and cats (28%) randomly collected on farms with a history of paratuberculosis (Beard et al. 2001; Palmer et al. 2005). However, in these studies and those in domestic dogs, there was no clear association of MAP isolation with gastrointestinal signs or histological lesions. When Kukanich, Vinasco and Scott (2013) compared intestinal biopsies of dogs with and without signs of gastrointestinal disease using PCR for detection of MAP, infection was confirmed in 19% of affected dogs and none of the controls. Although the previous report suggests (Glanemann et al. 2008) that carnivores can be infected with MAP, this is the first case in a dog in which infection was associated with granulomatous disease.

**FIGURE 5:**
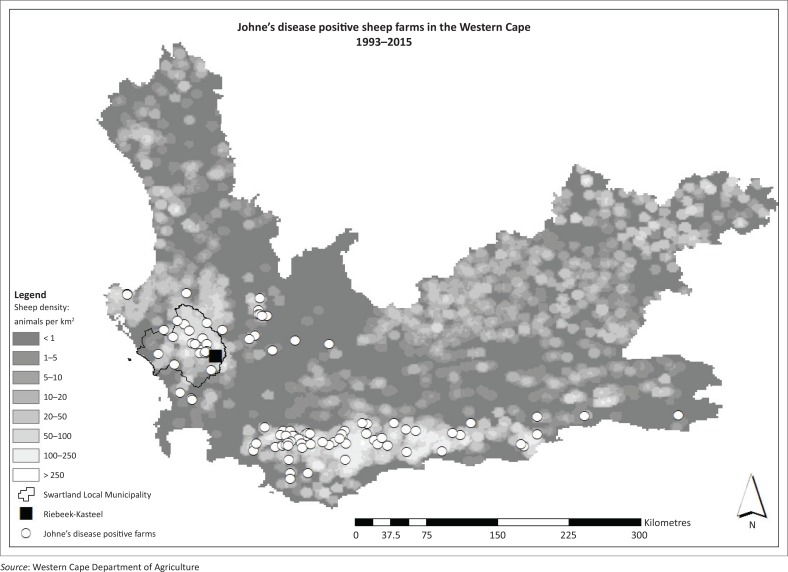
Map of Johne’s positive sheep farms in the Western Cape 1993–2015.

MAP infection in sheep has been documented in South Africa, particularly the Western Cape, where the dog in this case was located (Michel & Bastianello 2000). Since 2007, approximately 20% of sheep farms reported to be infected with Johne’s disease in the Western Cape occurred in the high-density sheep farming area of the Swartland local municipality (Figure 5).

Because transmission occurs through the faecal–oral route, susceptible animals in shared environments may be potentially infected (Daniels et al. 2003; Morkresh, Czuprynski & Butler 1989; Whittington et al. 2000). A potential exposure to MAP was revealed by the history of this dog. Originally from a breeder in the Swartland local municipality, the puppy moved into town with new owners (S. Davey [Western Cape Department of Agriculture] pers. comm., 26 March 2016). However, since the age of 6 months, the dog was taken on walks in a nearby sheep grazing area, with a history of Johne’s disease. Owners reported that the dog ingested animal faeces during walks, although this was not specified as sheep dung. It is unknown whether the dog had the opportunity to hunt or scavenge rodents in this area. Regardless of the exact source, this dog had possible repeated exposure to an infected environment for as long as 18 months. Although MAP infection in carnivores is rare, it should be included in the differential diagnosis of a dog with abdominal lymphadenopathy, splenomegaly or granulomatous ileitis.

Presence of granulomatous inflammation or Ziehl–Neelsen positive bacilli in tissues should increase suspicion of mycobacterial infection. Definitive diagnosis requires identification of MAP organisms by culture or PCR of tissues. As the growth of MAP requires specialised media, it is imperative that samples are submitted to laboratories with expertise in the culturing of mycobacteria (Whittington et al. 1999).

Dogs are unlikely to be a source of infection of MAP. Despite controversial association between MAP and Crohn’s disease, the zoonotic risk associated with paratuberculosis in companion animals is very low (Daniels et al. 2003; Hermon-Taylor 2001).

There is little information regarding treatment of paratuberculosis in animals (http://www.johnes.org/anitmicro/index.html; Bryant et al. 2012). Although a 2-month course of antibiotics resulted in improvement of clinical signs, there was no appreciable change in the MAP infection, which eventually resulted in euthanasia because of disease progression. Prognosis is guarded in cases of MAP because there is no known effective treatment in animals.

This case demonstrates the importance of a thorough history and diagnostic workup for detecting mycobacterial infections in domestic dogs.
